# Meet Our Editorial Board—*Engineering in Life Sciences*. An Interview With Michael Zavrel

**DOI:** 10.1002/elsc.70023

**Published:** 2025-04-14

**Authors:** Paul Trevorrow, Michael Zavrel

**Affiliations:** ^1^ Wiley The Atrium Southern Gate Chichester UK; ^2^ Technische Universität München Straubing Germany


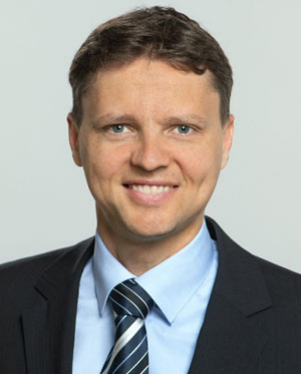


Professor Zavrel studied Chemical Engineering at TUM with a semester abroad at the University of California in Santa Barbara, USA. After completing his diploma thesis at Roche Diagnostics, he did his doctorate at the Chair of Biochemical Engineering at RWTH Aachen University. From 2008 to 2022, he worked in industrial research and development at Süd‐Chemie and Clariant, including as Head of Development & Biomanufacturing and as Site Manager. In 2022, Prof. Zavrel was appointed to the professorship for bioprocess engineering at the TUM.

## Would You Briefly Explain What Your Research Group Is Studying?

I am a professor of bioprocess engineering at the Technical University of Munich, specializing in the utilization of renewable resources. My research focuses on developing bioprocesses that use enzymes and microorganisms to convert agricultural residues, such as wheat straw and sugar cane bagasse, into products like biopolymers, biofuels, and bio‐based chemicals. Previously, I spent a significant period in industry, working at Süd‐Chemie and later at Clariant, where I held several positions, including the head of bioprocess development and biomanufacturing.

Although I did not initially plan to return to academia, an unforeseen opportunity arose, leading me to apply for this new professorship. Starting from scratch, I have been building my team and setting up the necessary equipment. My experience in scaling up processes from laboratory to industrial scale is seldom among those who have remained solely in academia. This expertise allows me to contribute significantly to the academic environment by focusing on technology transfer from the lab to larger scales, including cost calculations and life cycle assessments.

I am honored to be a member of the editorial board and look forward enthusiastically to participating actively in this role. My collaboration with partners, demonstration plants, and pilot plants ensures that my work remains practically oriented, bridging the gap between basic science and large‐scale industrial applications.

## What Are Your Views on the Future of Bioprocess Engineering?

I aim to contribute to trends such as using renewable materials over fossil‐based ones, ensuring these materials don't compete with the food chain. With the growing population and limited agricultural space, it's crucial to utilize all parts of plants efficiently. For example, using lignocellulosic parts for chemical processes.

I'm also concerned about the increasing presence of micro and nano plastics, which recycling cannot fully address. Developing biodegradable biopolymers that do not persist in the environment is essential.

Additionally, I focus on leveraging digitalization and artificial intelligence for better fermentation control through pattern recognition and continuous improvement.

## What Are Your Favorite Pastimes Outside of Research?

That's a good question. I have a family with two young children, aged eight and eleven, which occupies most of my personal time. I greatly enjoy spending time with them. Additionally, I engage in running and hiking, activities that my children increasingly participate in as well. These activities provide a beneficial break from work‐related matters.

Since becoming a professor, my personal time has been devoted to settling my family into our new location and managing organizational tasks related to establishing my new professorship. Consequently, my private time is largely dedicated to family.

## What Is the First Thing You Do When You Wake Up?

When I wake up, my alarm clock always rings. Unlike years ago, when I could sleep in on weekends, now I'm usually the first one awake. On Saturday morning, I go to the bakery to get bread and “Brezen” for breakfast, which my Kids like.

## Data Availability

Data sharing is not applicable to this article as no new data were created or analyzed in this study.

